# Supplementary material for: High performance implementation of the hierarchical likelihood for generalized linear mixed models: an application to estimate the potassium reference range in massive electronic health records datasets
Source: BMC Med Res Methodol. 2021 Jul 24;21:151. doi: 10.1186/s12874-021-01318-6 (PMC8310602; doi:10.1186/s12874-021-01318-6)
Supplement: Supplementary file 1 — Additional file 1. (Microsoft Word Document, Office 365 .docx). Supplementary Methods and Summary Statistic Data for the simulated datasets. [file 12874_2021_1318_MOESM1_ESM.docx]

# Supplementary Methods

## Simulation of Artificial Repeated Measures Electronic Health Record Datasets with Arbitrary Crossed Random Effect Design Matrix Structure

In this section we discuss how we simulated datasets with two random effects that could be either nested or crossed to an arbitrary extent. For Electronic Health Records (EHR), we can conceptualize these random effects to arise from *inter-individual variability* , i.e. random variation between patients that is not adequately captured by fixed covariates and *inter-healthcare-facility* variation, i.e., random variation due to facility policies and procedures. In a different , e.g., educational setting, one could postulate variability due to students and teachers/curricula. Real world datasets of this nature are never balanced since one would never have the same number of observations per cluster; furthermore, they will most often demonstrate an unbalanced crossed structure, i.e., not every lower-level unit will contribute to the same number of upper-level units. By the latter we mean, that the observations from a cluster at the lower level of the hierarchy may provide information for more than one clusters at a higher level of the hierarchy. For example, a patient (lower-level cluster) may visit more than healthcare facilities (higher level cluster), or a student may be taught by more than one educator. However, not every patient or student will interact with all doctors or teachers. To conform to our healthcare focus, we will refer to the lower (higher) level of clusters as “individual patients” (IP) and “healthcare facilities” (HCF) respectively. We devised a hierarchical simulation strategy to generate arbitrary datasets with a partially crossed structure:

1. We fix the number of IP ($N_{IP}$) and HCF ($N_{HCF}$) respectively.
2. For $j=1,\ldots, N_{IP}$ , we simulated observations (“visits”) ($N_{j}^{v}$) from a truncated negative binomial distribution. The latter was parameterized by a mean parameter ($\mu_{v}$), a size parameter ($\phi_{v}$), a lower ($m_{v}$) and an upper bound ($M_{v}$), i.e.,

$$N_{j}^{v} \sim NB\left( \mu_{v},\phi_{v} \right)T\left( m_{v},M_{v} \right)$$

The corresponding non-truncated negative binomial has mean $\mu_{v}$ and variance $\mu_{v}+{\mu_{v}^{2}}/{\phi_{v}}$. This specification allows us to vary both the expected number of repeated measures per individual ($\mu_{v}$), and control how unbalanced the dataset ($\phi_{v}$) will be, while limiting extreme events (number of repeated observations too high, or too low) that may arise during simulations by using the two bounds.

1. For $j=1,\ldots, N_{IP}$ , we simulated the number of distinct HCF ($N_{j}^{f}$) the jth patient visited from a truncated Poisson distribution. The latter was parameterized by a rate parameter ($\lambda_{f}$), a lower ($m_{f}$) and an upper bound ($M_{f}$), i.e.,

$$N_{j}^{f}\sim Poisson\left( \lambda_{f} \right)T\left( m_{f},M_{f} \right)$$

The rate parameter controls the propensity of each lower unit to be associated with numerous upper-level units, while the use of bounds allows one to further tune this propensity. In the case that $m_{f}>0$and $M_{f}$ < 2, this specification allows the user to simulate nested random effects.

1. For $j=1,\ldots, N_{IP}$ , we simulated the probability that the jth individual visits one of the $N_{j}^{f}$ facilities, as well as the number of visits to each of these facilities in hierarchical fashion:
   1. Sample positive quantities $\alpha_{1},\ldots,a_{N_{HCF}} \sim Lognormal\left( \mu_{\alpha},\sigma_{\alpha} \right)$
   2. Sample uniformly and without replacement $z_{1},\ldots,z_{N_{j}^{v}}$ from the set $\alpha_{1},\ldots,a_{N_{HCF}}$
   3. Calculate the probability that the jth individual will visit each of the $1,\ldots, N_{j}^{f}$ as:

$$p_{i,j}=\frac{z_{i}}{\sum_{k=1}^{N_{j}^{f}} z_{k}}$$

   4. Sample the number of visits $n_{1,j},\ldots,n_{N_{j}^{v},j}$ from the multinomial distribution with total sample size $N_{j}^{f}$ and vector of probabilities $p_{i,j}$, $i=1,\ldots, N_{j}^{f}$:

$$n_{1,j},\ldots,n_{N_{j}^{v},j} \sim Multinomial\left( N_{j}^{f};p_{1,j},\ldots,p_{N_{j}^{v},j} \right)$$

The three nesting scenarios considered in this paper were generated by the parameters shown in ***Table 1***

| **Parameters** | **Nested** | **Part Crossed** | **More Crossed** |
| --- | --- | --- | --- |
| $m_{v},M_{v}$ | 1 , 10 | 1 , 10 | 1 , 10 |
| $\mu_{v},\phi_{v}$ | 7.74 , 0.575 | 7.74 , 0.575 | 7.74 , 0.575 |
| $m_{f},M_{f}$ | 0.0 , 1.1 | 0, 0.1 + $N_{HCF}$ | 0, 0.1 + $N_{HCF}$ |
| $\lambda_{f}$ | 0.25 | 1.0 | 2.25 ($N_{IP}=100$) or 25.25 ${(N}_{IP}>100$) |
| $\mu_{\alpha},\sigma_{\alpha}$ | 3 , 0.2 | 3 , 0.2 | 3 , 0.2 |

***Table 1*** *Parameters used to simulate datasets with various levels of crossing.*

## Simulation of Covariates and Design Matrices

Covariates for the Poisson regression models were simulated as independent draws as follows: simulated age, potassium, eGFR were drawn from truncated normal distributions:

$$Age_{j} \sim Normal\left( \mu_{age},\sigma_{age} \right)T\left( m_{age},M_{age} \right)$$

$$K_{j} \sim Normal\left( \mu_{K},\sigma_{K} \right)T\left( m_{K},M_{K} \right)$$

$${eGFR}_{j} \sim Normal\left( \mu_{eGFR},\sigma_{eGFR} \right)T\left( m_{eGFR},M_{eGFR} \right)$$

The potassium (K) level was further “randomized” between repeated measures from the same individual. In particular, the K level from the lth visit of the jth individual was drawn from a normal distribution, with the same mean as $K_{j}$ and standard deviation that was proportional to $K_{j}$:

$$K_{l,j} \sim Normal\left( K_{j},p_{k}K_{j} \right)$$

Gender and the Charlson Comorbidity Index (CCI), were drawn from a binomial and a truncated negative binomial distribution respectively:

$Gender_{j} \sim Binomial\left( 1;p_{gender} \right)$

$$CCI_{j} \sim NB\left( \mu_{CCI},\sigma_{CCI} \right)T\left( m_{CCI},M_{CCI} \right)$$

The Length of Stay of the lth visit of the jth patient was drawn from a log normal distribution:

$$LOS_{l,j} \sim Lognormal\left( \mu_{LOS},\sigma_{LOS} \right)$$

The use of bounds during covariate simulations allowed the user to limit covariate values to realistic ranges.

The parameters used to generate the fixed design matrices for the simulations are shown in ***Table 2***:

| **Parameters** | **Value** |
| --- | --- |
| $\mu_{age},\sigma_{age}$ | 58, 12 |
| $m_{age},M_{age}$ | 18, 100 |
| $\mu_{K},\sigma_{K}$ | 4.1 , 1 |
| $m_{K},M_{K}$ | 2 , 8 |
| $p_{k}$ | 0.05 |
| $\mu_{eGFR},\sigma_{eGFR}$ | 82 , 28 |
| $m_{eGFR},M_{eGFR}$ | 15 , 120 |
| $p_{gender}$ | 0.44 |
| $\mu_{CCI},\sigma_{CCI}$ | 0.98 , 0.55 |
| $m_{CCI},M_{CCI}$ | 0 , 29 |
| $\mu_{LOS},\sigma_{LOS}$ | -0.1483469, 1.413642 |

***Table 2*** *Parameters used to simulate fixed effect covariate values.*

After simulating eGFR, potassium value and age for each patient, these covariates entered the model as natural splines with the boundary and interior knots shown in :

| **Covariate** | **Boundary Knots** | **Interior knots** |
| --- | --- | --- |
| Age | 18, 100 | 50 , 66 |
| Potassium | 2 , 8 | 3 , 5 |
| eGFR | 15 , 120 | 50 , 90 |

***Table 3*** Position of knots used for covariates that entered the simulations through splines.

## Simulation of Sparse Poisson Outcomes

Poisson outcomes were simulated from the design matrix of the fixed and random effects in a hierarchical fashion:

1. Simulate datasets with different number of individuals and HCF (either $N_{IP}=100$ and $N_{HCF}=5$ or $N_{IP}=1000$ and $N_{HCF}=50$) using the parameters shown in ***Table 1***. Two hundred datasets were generated for the six combinations of dataset size (number of individuals and HCF) and degree of crossing (a total of 1,200 simulated datasets).
2. Simulate fixed effects covariates using the parameters and spline knot positions in ***Table 2*** and ***Table 3*** respectively.
3. The contribution of the fixed effects to the linear predictor was calculated by multiplying the matrix of the fixed effects with the vector of the fixed effects coefficients. The fixed effect coefficients used for all three scenarios are listed below (covariates entering the model as splines have more than one coefficient associated with them:
   $\boldsymbol{\beta}_{age}\boldsymbol{=}\{1.0 ,2.0 ,1.5\}$, $\boldsymbol{\beta}_{K}\boldsymbol{=}\{1.5, -1.1, 2.2\}$, $\boldsymbol{\beta}_{eGFR}\boldsymbol{=}\{1.0, 0.2, 0.12\}$, $\beta_{CCI}=0.15$, $\beta_{Gender}=0.26$ . The coefficient associated with the intercept was used to scale the number of counts with the size of the dataset and varied according to the number of HCF and IP:
   $\beta_{N_{HCF}=5,N_{IP}=100}^{0}=-4.5$, $\beta_{N_{HCF}=5,N_{IP}=1000}^{0}=-5.5$
4. The contribution of the random effects to the linear predictor was derived by cross classifying observations according to HCF and IP. For $j=1,\ldots, N_{IP}$ and $i=1,\ldots, N_{HCF}$ we simulated independent random effects: $u_{i}^{HCF}\sim Normal(0,\sigma_{HCF}$) and $u_{j}^{IP}\sim Normal(0,\sigma_{IP}$). These terms were added to the corresponding fixed effect term and then exponentiated to compute a rate parameter for each observation. The standard deviation of the random effects was set to $\sigma_{HCF}=0.5$ and $\sigma_{IP}=1.0$.
5. The rate parameter computed in the previous step was then used to draw a Poisson distribution that was truncated to lie in the interval 0 to 1.

## Simulation of Binary Observations

The hierarchical simulation for the binary outcomes proceeded as:

1. Simulate nested and crossed datasets using the parameters shown in ***Table 3*** for three different dataset sizes : $N_{IP}=100$ and $N_{HCF}=5$ or $N_{IP}=1000$ and $N_{HCF}=50$ or $N_{IP}=10000$ and $N_{HCF}=50$
2. Sample random effects from their respective distributions. There are two random effects : one at the IP and one at the HCF level: $u_{i(l)}^{HCF}\sim Normal(0,\sigma_{HCF}$),$u_{j}^{IP}\sim Normal(0,\sigma_{IP}$).
3. The logit of the outcome of the lth repeated measure of the jth individual , which occurred in the $i(l)$th HCF was computed as:

$\mathrm{logit}\left( p_{l,j} \right)=\beta_{N_{HCF},N_{IP}}^{0}+u_{j}^{IP}+u_{i(l)}^{HCF}$

1. The binary outcome was simulated as : $I_{j,j} \sim Bernoulli\left( \mathrm{invlogit}\left( p_{l,j} \right) \right)$.

We considered two different combinations of random effect standard deviations (“less variable” and “more variable”) for each of the nine combinations of dataset size and crossing. The intercept values (which control the sparsity of the outcomes) and the standard deviations are shown in ***Table 4***.

| **Dataset Size** | **Less Variable** | **More Variable** |
| --- | --- | --- |
| $N_{IP}=100$  $N_{HCF}=5$ | $\beta_{N_{HCF}=5,N_{IP}=100}^{0}=-5.5,$  $\sigma_{HCF}=0.5,$  $\sigma_{IP}=1.0$ | $\beta_{N_{HCF}=5,N_{IP}=100}^{0}=-5.5,$  $\sigma_{HCF}=0.5,$  $\sigma_{IP}=2.5$ |
| $N_{IP}=1000$  $N_{HCF}=50$ | $\beta_{N_{HCF}=5,N_{IP}=1000}^{0}=-7.5$  $,\sigma_{HCF}=0.5$  $, \sigma_{IP}=1.0$ | $\beta_{N_{HCF}=5,N_{IP}=1000}^{0}=-7.5,$  $\sigma_{HCF}=0.5,$  $\sigma_{IP}=2.5$ |
| $N_{IP}=10000$  $N_{HCF}=50$ | Not considered | $\beta_{N_{HCF}=5,N_{IP}=10000}^{0}=-9.5,$  $\sigma_{HCF}=0.5,$  $\sigma_{IP}=2.5$ |

***Table 4*** Parameters used to generate binary outcomes.

Two hundred datasets were generated for each of the fifteen combinations of dataset size, degree of crossing, and variability considered for a total of 3,000 datasets.

**Supplementary Figure 1** Distribution of repeated measures per individual patient (IP), and number of distinct health care facilities (HCF) visited by each IP in the simulated datasets for the three different nesting scenarios considered in the paper.

**
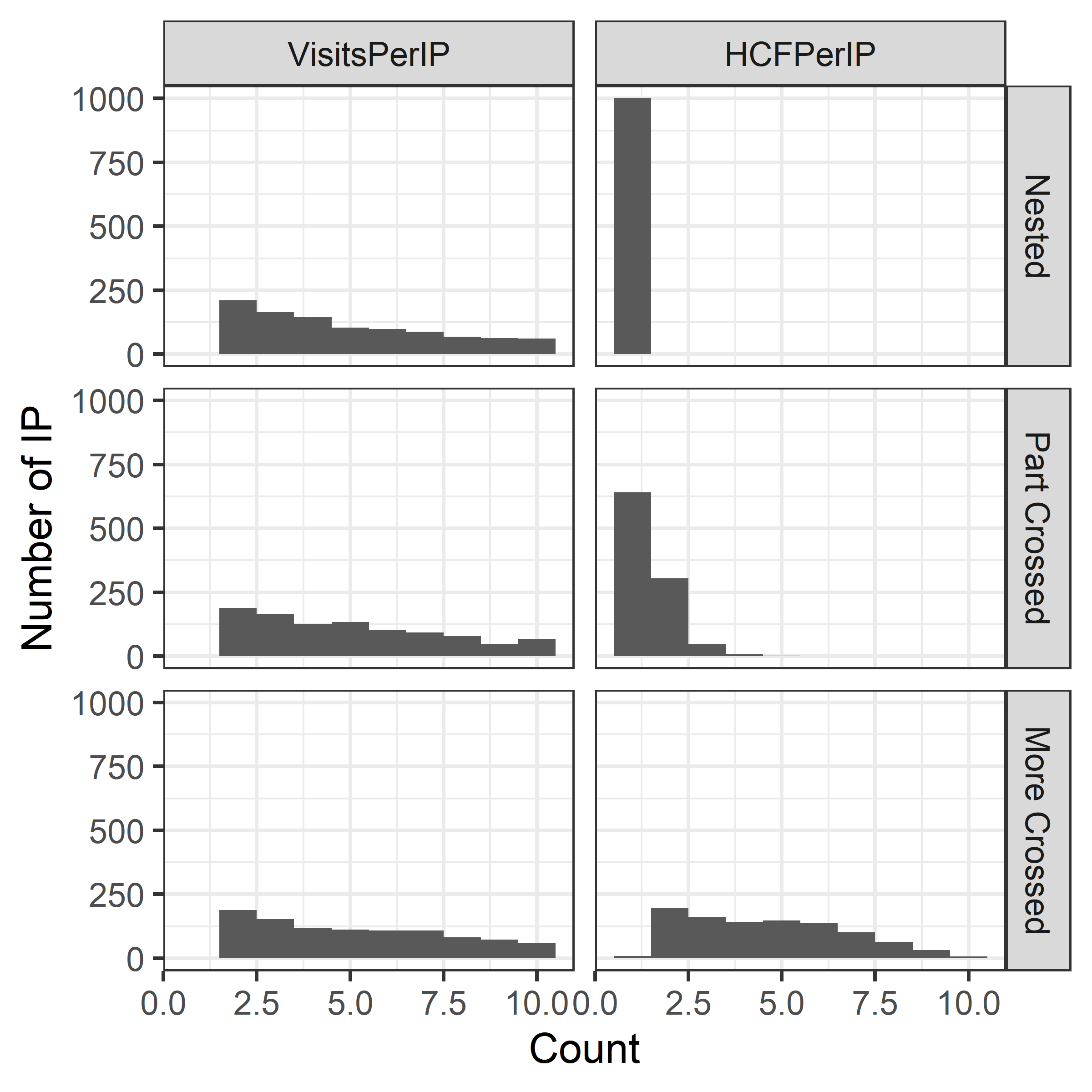
**

**Supplementary Figure 2** Distribution of repeated measures per health care facility (HCF) and individual patient (IP) within each HCF in the simulated datasets for the three different nesting scenarios considered in the paper.

**
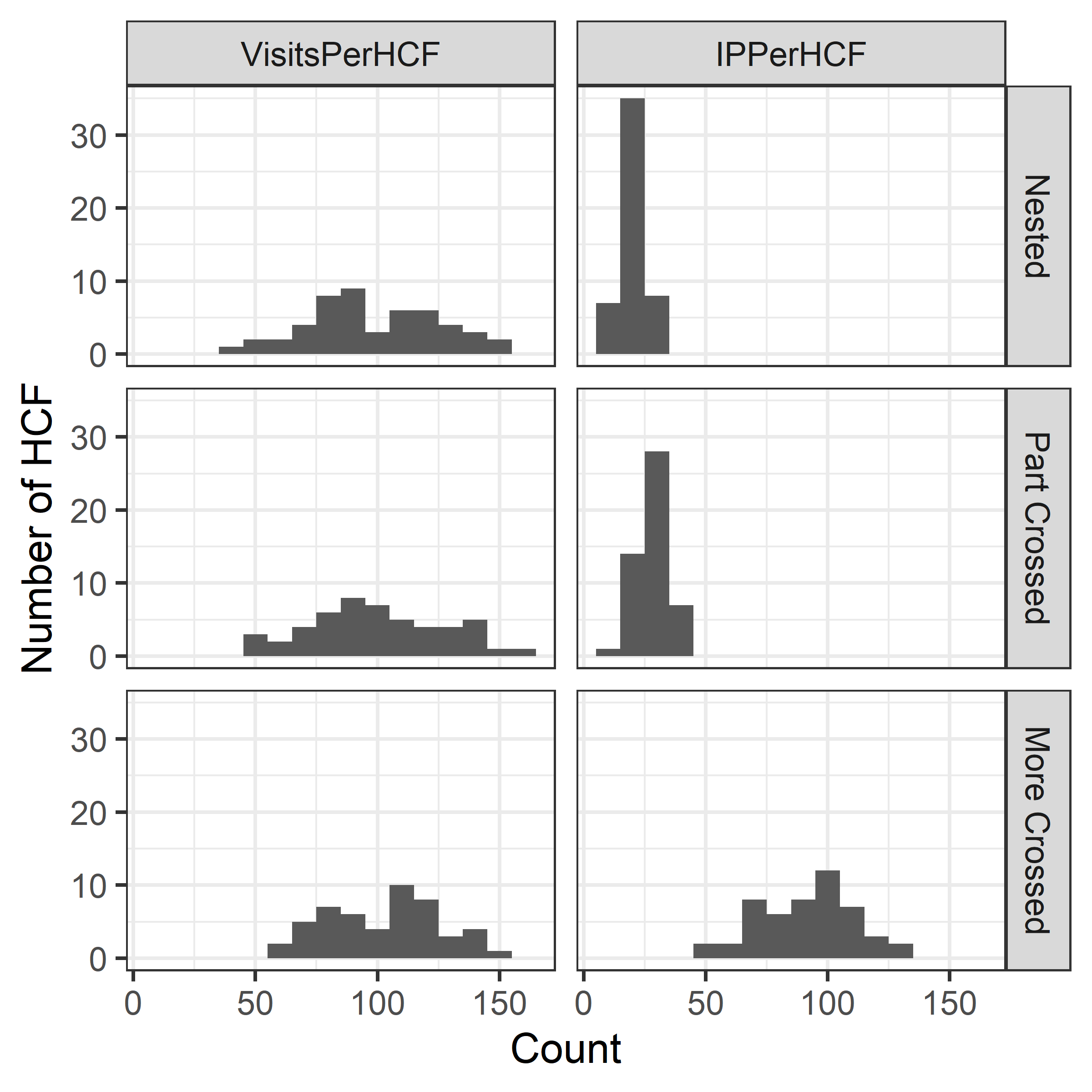
**

**Supplementary Figure 3** Event rates in the simulated Poisson, “less” and “more” variable binary (binomial) datasets according to nesting scenario and number of individual patients

**
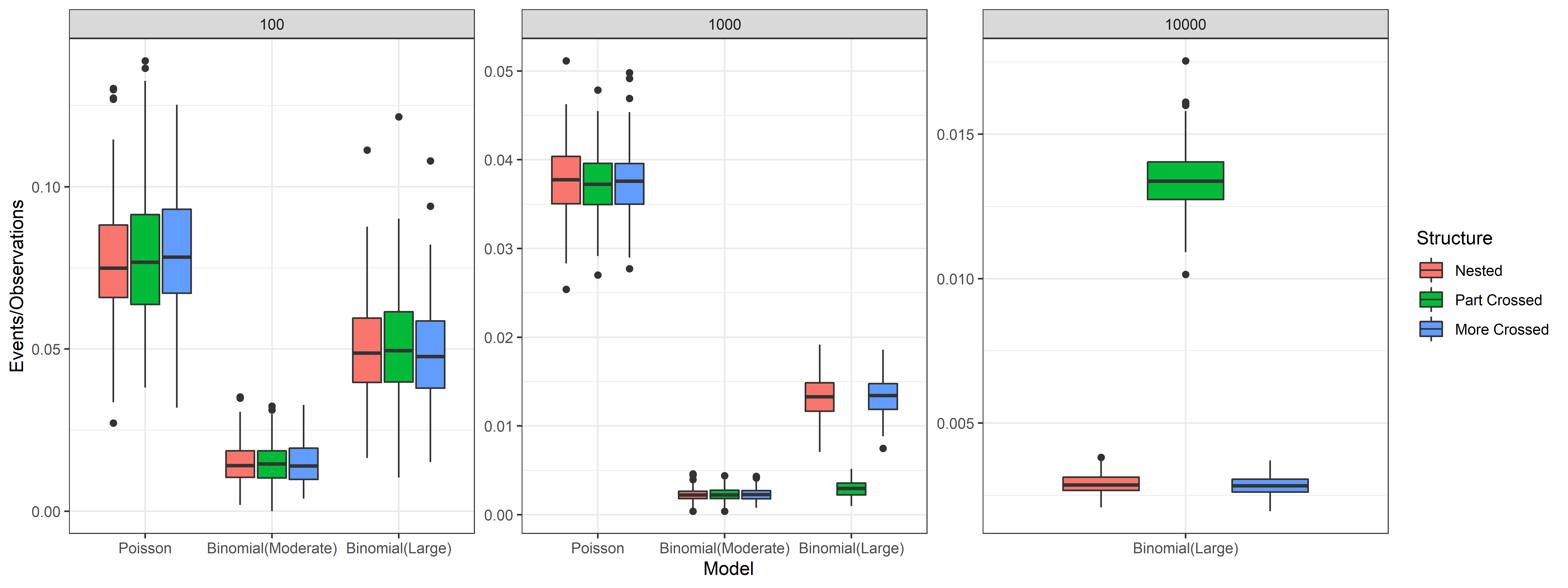
**
