# Supplementary figures and images for: High performance implementation of the hierarchical likelihood for generalized linear mixed models: an application to estimate the potassium reference range in massive electronic health records datasets
Source: BMC Med Res Methodol. 2021 Jul 24;21:151. doi: 10.1186/s12874-021-01318-6 (PMC8310602; doi:10.1186/s12874-021-01318-6)

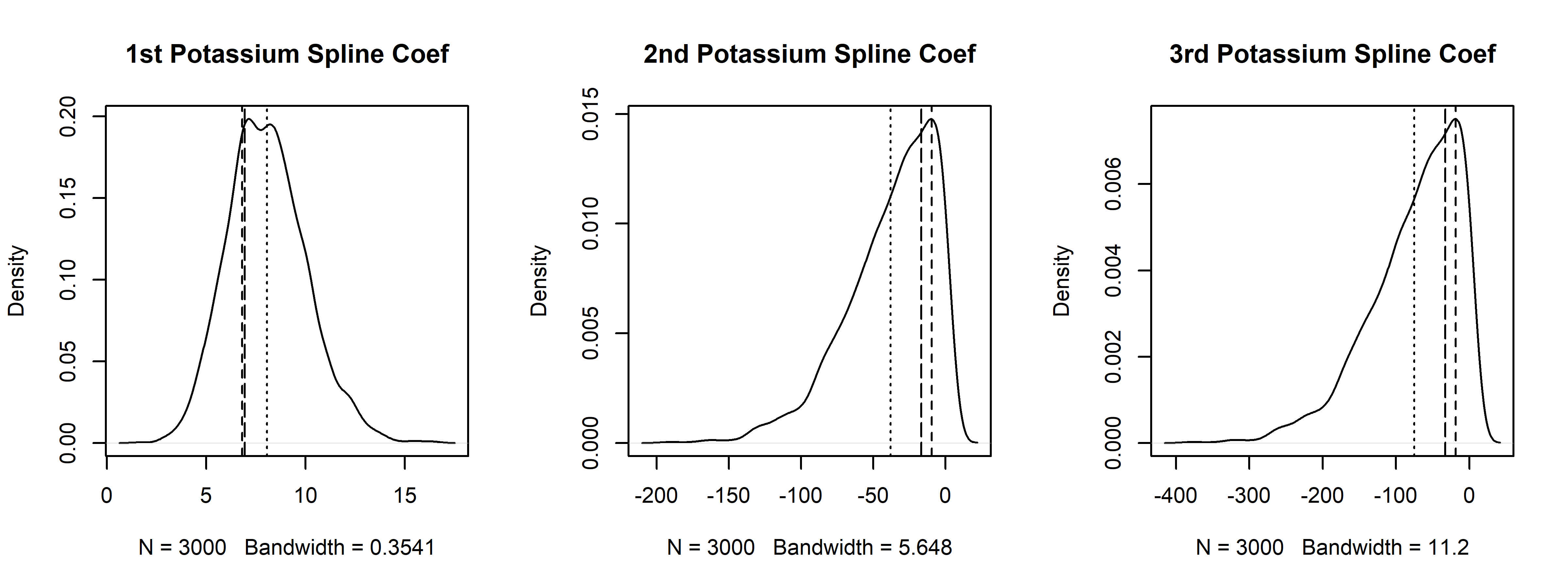

Supplement: Supplementary file 2 — Additional file 2. (Portable Networks Graphics image .png). Non-parametric kernel density estimates of the posterior marginal density of the three spline coefficients (solid curve) against the MCMC posterior mean (dotted vertical line), the AGH9 based estimated (long dash) and the h-lik estimate (dashed vertical line). [file 12874_2021_1318_MOESM2_ESM.png]
